# Developing a CAMHS Hub Crisis Management Service – the First Six Months

**DOI:** 10.1192/bjo.2024.490

**Published:** 2024-08-01

**Authors:** James McDonald, David Columb, Laura Moriarty, Clare Verdon, Katie Murphy

**Affiliations:** Lucena Clinic, Dublin, Ireland

## Abstract

**Aims:**

This poster will:

1. Describe the establishment of an acute crisis management service within a Child and Adolescent Mental Health Service (CAMHS) in the Republic of Ireland.
2. Summarise clinical activity during the first six months of the service and qualitative feedback from service users and clinicians on their experience of the service.

**Methods:**

In December 2021 the Republic of Ireland Health Service Executive approved the roll out of acute crisis management services for CAMHS – known as Hubs, with a remit to provide intensive brief interventions to support young people experiencing acute Psychiatric crises. Multiple weekly appointments are provided in clinic, at home or via telehealth.

The Lucena Clinic CAMHS – based in Counties Dublin and Wicklow, was chosen as a pilot site. Staff were recruited in January 2023 consisting of:

1. FTE Consultant Child and Adolescent Psychiatrist
2. Candidate Advance Nurse Practitioner
3. Senior Social Worker
4. Senior Occupation Therapist
5. Administrator

A multi-disciplinary Steering Group was established with a view to planning clinical programs, ensuring safety and governance, procuring resources and embedding service evaluation.

The service went live in May 2023. Clinical data was gained from data entry to the service Electronic Patient Record.

Qualitative feedback was gained from service users using post-discharge questionnaires and from clinicians using semi-structured interview.

**Results:**

1. Between May and December 2023 the Hub received 61 referrals and accepted 35.
2. 27 of those accepted were new referrals to the service.
3. Patients received an average of 27.1 hours of clinical intervention during their admission.
4. Shortest admission was 10 hrs, the longest 66.5 hrs.
5. 6 young people were seen at home, totalling 41 visits.
6. 24 young people were discharged to CAMHS, 2 to GP, 2 to the clinic's Day Program, 3 required in-patient admission.

Service user feedback was positive with families highlighting ease of access to the service, intensity of intervention and a friendly environment as positives.

One parent remarked that they did not feel the Hub was the right setting for their child's care.

Clinician feedback highlighted the Hub as a positive support for community CAMHS with rapid access to intervention and communication from the Hub team mentioned as positives. One drawback identified was the intensity of intervention setting an unrealistic expectation for ongoing care.

**Conclusion:**

The Hub appears a welcome addition to CAMHS with positive feedback from service users and clinicians. Ongoing development phase and evaluation is required.

